# Proteomic and Metabolomic Profiling in Soft Tissue Sarcomas

**DOI:** 10.1007/s11864-022-00947-3

**Published:** 2022-02-16

**Authors:** Madhumeeta Chadha, Paul H Huang

**Affiliations:** Division of Molecular Pathology, The Institute of Cancer Research, 15 Cotswold Road, Sutton, SM2 5NG UK

**Keywords:** Sarcoma, Proteomics, Metabolomics, Biomarkers, Drug discovery

## Abstract

Advances in proteomic and metabolomic technologies have accelerated our understanding of multiple aspects of cancer biology across distinct tumour types. Here we review the current state-of-the-art in the use of proteomics and metabolomics in soft tissue sarcomas. We highlight the utility of these Omics-based methodologies to identify new drug targets, synthetic lethal interactions, candidate therapeutics and novel biomarkers to facilitate patient stratification. Due to the unbiased and global nature of these profiling methods to assess the levels of protein expression, post-translational modifications such as phosphorylation and glycosylation as well as key metabolites, many of these findings have broad applications not just in specific histotypes but across multiple STS subtypes. Specific examples of proteomic and metabolomic findings that have led to the development of early phase clinical trials of investigational agents will be discussed. While promising, the use of these technologies in the study of sarcoma is still limited, and there is a need for further research in this area. In particular, it would be important to integrate these approaches with other Omics strategies such as genomics and epigenomics as well as implement these tools alongside clinical trials in order to maximize the impact of these tools on our biological understanding and treatment of this group of rare diseases of unmet need.

## Introduction

Sarcomas are a group of rare neoplasms originating from the malignant transformation of primary, multipotent mesenchymal stem cells which differentiate into bone, muscle, adipose and cartilage tissues [1,2]. They are primarily divided into two groups: bone sarcomas and soft tissue sarcomas (STS) [3]. While sarcomas can originate anywhere in the body, the most common site of occurrence is the extremities, consisting of 60% of all STS. The prevalence of STS varies significantly with age, accounting for about 7–15% of all paediatric and 1% of all adult malignancies, respectively [4]. STS consist of at least 80 histologic subtypes, with the most common ones being liposarcoma (LPS), leiomyosarcoma (LMS) and undifferentiated pleomorphic sarcoma (UPS) [1,5]. The heterogeneity of STS is further highlighted by the variable clinical behaviour displayed by each subtype [6].

Treatment of localized primary STS consists of surgery either with or without radiation [7]. Around 50% of patients relapse, often with distant metastasis [8]. In the case of locally advanced or metastatic STS, the first line of treatment has been anthracycline monotherapy or in combination with ifosfamide [9–11]. Following the failure of first-line therapy, there is a limited number of chemotherapy and targeted agents in the therapeutic arsenal including trabectedin, eribulin and pazopanib. However, the survival outcomes of STS patients remain poor with <10% of patients with advanced disease surviving beyond 5 years [12]. New agents are urgently required for this patient population of unmet need. While advances in genomics have led to new discoveries about the biology of STS, including the discovery of new fusion genes, new molecular subgroups within histological subtypes and prognostic and predictive classifiers, this remains only one facet of the disease [13–15]. The use of additional Omics-based technologies including proteomics and metabolomics will provide a holistic overview of the molecular mechanisms of disease which may open the door to new drug targets, unravel novel tumour cell vulnerabilities and aid biomarker discovery in STS. In this review, we will discuss the latest advances and provide an update on the use of proteomics and metabolomics in STS.

## Proteomics in soft tissue sarcomas

Proteomics represents the entire complement of proteins within the cell and includes post-translational modifications (PTMs) and protein-protein interactions [16,17]. Given that the vast majority of approved and investigational drugs target proteins and not genes, this approach can directly facilitate mining of existing and new drug targets for clinical translation [18]. Furthermore, given the latest developments in next-generation drug modalities such as protein degraders like proteolysis targeting chimeras (PROTACs), as well as immune-oncology agents that target cell surface proteins, the use of this technology will ensure that sarcoma research is at the forefront of next-generation drug discovery efforts [19,20]. However, unlike genomic technologies, there is no one standard pipeline for proteomics analysis. Proteomic tools can encompass multiple approaches including mass spectrometry and protein array technologies. Furthermore, there are technical challenges associated with measuring the plethora of PTMs in the proteome, the vast dynamic range of protein abundance and diverse physiochemical properties of amino acids [21]. Here we will discuss the latest proteomic studies in sarcomas.

One of the earliest large-scale proteomics studies in STS was performed by The Cancer Genome Atlas (TCGA) Research Network which carried out a multi-platform molecular analysis of 6 histological subtypes [22]. Here the authors employed the reverse phase protein array (RPPA) technology on 173 samples. Utilizing 192 antibodies representing different oncogenic pathways within tumour cells, the authors identified diverse molecular features of LMS when compared to other subtypes, which was characterized by a lower activity of apoptotic pathways alongside higher phosphoinositide 3-kinase (PI3K) pathway activity. More recently, our laboratory has used data-independent mass spectrometry (DIA-MS) to quantify 2900 protein across 36 STS cases spanning 4 histological subtypes [23•]. Analysis of formalin-fixed paraffin-embedded (FFPE) tissue identified a subgroup of STS cases of mixed histological subtypes that harboured a distinct proteomic signature which was associated with worse survival outcomes compared to the rest of the cohort. While the cohort is small, this study highlights the potential for proteomics to identify candidate prognostic biomarkers.

Two recent studies have sought to integrate genomics and proteomics in order to extract useful biological information about two histological subtypes, namely UPS and rhabdomyosarcoma (RMS). Toulmonde et al. performed integrative genomics and proteomics (DIA-MS) to categorize UPS clinical specimens into subgroups with distinct immune phenotypes, clinical outcomes and therapeutic sensitivities [24•]. The authors demonstrate that the “immune-low” subgroup is sensitive to FGFR inhibition and speculate that the “immune-high” subgroup may be amenable to immune checkpoint inhibitor therapy. In another study, Steward et al. performed a multi-omics study to identify vulnerabilities in RMS that can be targeted clinically [25••]. There are two RMS subtypes, translocation positive alveolar RMS (ARMS) and fusion negative embryonic RMS (ERMS), with ERMS having relatively good prognosis. However, a subset of ARMS are translocation negative and share molecular features with ERMS. Multi-omics genomic and epigenomic profiling was carried out in RMS orthotopic patient-derived xenografts (OPDX) generated from recurrent patient specimens. This included whole genome bisulphite sequencing, RNA sequencing and chromatin immunoprecipitation sequencing (ChIP-seq), and data was compared to normal tissue sharing the same lineage such as skeletal muscle, myoblasts and myotubes as controls. The authors also used isobaric labelling coupled to mass spectrometry to measure the proteome and phosphoproteome of these OPDX models alongside human myoblasts and myotubes. This led to identification of 16,523 proteins and 12,653 phosphosites with weighted gene co-expression analysis identifying six groups of proteins and phosphoproteins differentially expressed in ARMS, ERMS and developing muscle. After integrating data obtained from multiple platforms, co-expression clustering revealed 4 integrated clusters (IPCs). IPC1 contained genes/proteins upregulated in tumours relative to myoblasts, IPC2 contained those upregulated in ERMS compared to ARMS, IPC3 contained those specifically upregulated in ARMS and IPC4 contained those which were upregulated in myoblasts compared to tumours. The authors also identified therapeutically relevant pathways such as CDK4/6 and G2/M mitotic spindle checkpoint signalling which were dysregulated in RMS and successfully targeted these pathways in preclinical models.

In addition to measuring total protein levels, it is possible to quantitatively measure PTMs including glycosylation and phosphorylation using mass spectrometry. In a pioneering study by the Bovée laboratory, the authors used mass spectrometry imaging to measure the N-glycans in myxoid liposarcoma (MLS) [26•]. Given the intratumour heterogeneity inherent in patient specimens, spatial information can provide novel insights that conventional bulk Omic analysis might miss. To undertake mass spectrometry imaging analysis, a tumour microarray (TMA) was constructed from 32 patients that did not receive neoadjuvant therapy. The array consists of 141 cores obtained from 3 morphological areas of MLS tumours alongside 6 control cores obtained from various anatomical locations. The authors then compared the N-glycan content from different morphological areas and between different grades of tumours. The authors found that high-mannose-type glycans along with tri- and tetra-antennary complex N-glycans were significantly different in morphologically distinct areas and between various tumour grades. They show that an increase in high-mannose-type N-glycans and tri-antennary complex N-glycans (known as CA3 trait) is indicative of disease progression where patients with high abundance of CA3 trait display a significantly poorer prognosis compared to patients with low abundance.

Phosphorylation is one of the most prevalent, widely studied PTMs intimately involved in almost all cellular processes [27]. To gain a comprehensive overview of tyrosine phosphorylation (pY) signalling pathways in sarcoma cell lines, Bai et al. profiled 10 cell lines of different histologies including osteosarcoma, RMS, LMS, fibrosarcoma and Ewing’s sarcoma [28]. They identified 844 unique pY proteins corresponding to nearly 40% of the tyrosine kinome. The authors then selected several signalling proteins including multiple receptor tyrosine kinases (RTK) and their downstream kinases for validation studies by either deploying RTK arrays or western blots. Screening of sarcoma lines with a panel of inhibitors and RNA interference experiments that targeted key kinases revealed previously undescribed vulnerabilities across multiple subtypes. These included the identification of platelet-derived growth factor receptor alpha (PDGFRα) as an important target of RMS. More recently, Roger Daly’s laboratory has used a similar approach in 20 different sarcoma cell lines comprising angiosarcoma, gastrointestinal stromal tumour, Ewing’s sarcoma, RMS and synovial sarcoma [29]. They identified 654 pY proteins whose profiles are clustered into two major groups comprising adult sarcomas and paediatric/young adult sarcomas. Focusing on the synovial sarcoma subtype, they found several new RTK dependencies including ALK, PDGFRα and MET which were functionally tested in preclinical in vivo experiments and whose expression was shown to be elevated in a subset of synovial sarcoma patient specimens. These phosphoproteomic studies hold the promise for the identification of phosphoprotein biomarkers to enable patient stratification for kinase inhibitor-based targeted therapies.

## Metabolomics in soft tissue sarcomas

One key feature of cancer cells is metabolic adaption to meet the high demands of cell growth and division [30]. Metabolic reprogramming to utilize glucose and amino acids in a distinct manner from non-malignant cells is considered an important hallmark of cancer and was described first by Otto Warburg [31]. Under physiological conditions, normal quiescent cells channel mitochondrial oxidative phosphorylation (OXPHOS) to maximize energy production with minimum lactate production. Enhanced production of lactate by healthy cells is accounted for in anaerobic conditions. In contrast, malignant cells produce lactate regardless of oxygen availability by a process termed as “aerobic glycolysis” [32]. The study of the metabolome, a collection of small molecules involved in metabolic processes, is known as metabolomics [33]. Technological advancements in analytical techniques such as mass spectrometry, nuclear magnetic resonance and isotope tracing have redefined our ability to measure the metabolome and have led to new discoveries in the biology of STS [34]. From a therapeutic standpoint, targeting the metabolic pathways in tumours is an attractive strategy. While most tumours are genetically distinct, alterations in many oncogenes and tumour-suppressor genes induce a common metabolic phenotype. Targeting these shared characteristics may be considerably more advantageous than treatments based solely on individualized genetic profiles [35]. Also, given the strong association between metabolism and the immune microenvironment, targeting metabolic pathways to enhance immune cell function is also an emerging area of research and the focus of new therapies [36,37]. For instance, rapamycin is a common immunosuppressive drug which specifically inhibits mTOR, a key regulator of amino acid, nucleotide, fatty acid and glucose metabolism. Among its many effects on the immune system, it has been shown to enhance the survival of antigen-specific memory T cells [38]. While there are some sarcomas for which there are clear aberrations in metabolic pathways, most prominently in some bone sarcomas such as chondrosarcoma where ~50 % of patients harbour mutations in the isocitrate dehydrogenase (IDH) enzymes, the metabolic profiles and vulnerabilities of the majority of STS subtypes remain uncharacterised [39,40]. Building on studies in other cancer types, a more comprehensive understanding of the STS metabolome could lead to new agents for this disease of unmet need.

### Glycolysis

A recent study utilized several metabolomic techniques such as unbiased mass spectrometry and isotope tracing to demonstrate that the loss of fructose-1,6 bisphosphatase 2 (FBP2) is a key event during sarcoma progression and a potential therapeutic target [41••]. Glucose homeostasis maintained by glycolysis and gluconeogenesis plays an important role in tumour growth. An important rate-limiting step is irreversible hydrolysis of fructose-1,6-bisphosphate to fructose-6-phosphate and inorganic phosphate by the enzyme FBP. FBP has two isoenzymes; FBP1 is present in the liver and kidneys, while FBP2 has highest expression in skeletal muscle and other mesenchymal tissues. While the role of FBP1 in various carcinomas has been studied, the role of FBP2 in mesenchymal cancers such as STS has not been explored. Huangyang et al. showed that loss of FBP2 is a common feature seen across multiple STS subtypes and plays an important role in tumourogenesis. Using isotope labelling, the fate of metabolites derived from 1,2-^13^C glucose in the glycolytic pathway, tricarboxylic acid (TCA) cycle and pentose phosphate pathway was studied in 5 STS cell lines after FBP2 re-expression. The authors showed that FBP2 re-expression resulted in metabolic adaptation with a decrease in glucose uptake and conversion to lactate, a decrease in four TCA intermediates (citrate, α-ketoglutarate, fumarate and malate) and glutamate and aspartate. They also observed an increase in intermediates of other metabolic pathways such as amino sugars, erythrose-4-phosphate and gluconolactone. Gene set enrichment analysis of RNA-seq data from FBP2 expressing cells revealed reduced enrichment of gene signatures for known oncogenic pathways such as MYC targets, OXPHOS, G2/M checkpoint regulation and E2F targets. This study highlights the importance of FBP2 in STS biology and its pleiotropic impact on multiple key oncogenic pathways. Further studies are required to translate these preclinical findings into actionable therapeutics for evaluation in clinical trials.

### Amino acid metabolism

Another aspect of STS biology is amino acid metabolism. Several studies have identified vulnerabilities in glutamine, arginine and tryptophan metabolism in STS. Circulating glutamine is one of the most abundant amino acids and a vital source of nitrogen for biosynthetic reactions. It serves as a source of carbon to replenish the TCA cycle and produce glutathione and as a precursor for lipid and nucleotide synthesis [42]. Mitochondrial glutaminase (GLS) is a rate-limiting enzyme that converts glutamine to glutamate by glutaminolysis [43].

In one study, Lee et al. showed that glutamine metabolism is particularly important in UPS [44•]. The authors used two distinct autochthonous models of UPS, KP (driven by KrasG12D and loss of Trp53) and KPH2 (driven by KrasG12D, loss of Trp53 and loss of Epas1) which faithfully recapitulate the human disease. Tumours from KP, KPH2 and tissue from muscle that serves as a control were used for comparative metabolomic experiments. The authors showed that in KP and KPH2 tumours, glutamine-related metabolism was highly active as shown by an increase in glutamate, aspartate and asparagine abundance and a decrease in glutamine levels. Tumour cells derived from the mouse models as well as cell lines from multiple STS subtypes were cultured in glucose-free or glutamine-free media which demonstrated a critical dependency on glutamine with observed reduction in both cell proliferation and viability. As GLS converts glutamine to glutamate, expression of this enzyme was evaluated by IHC staining which showed that UPS, synovial sarcoma and LMS specimens had high levels of protein expression compared to human skeletal muscle and LPS. The authors performed elegant stable isotope labelling tracing and mass spectrometry experiments to show that glutamine anaplerosis promotes de novo nucleotide production to sustain cancer cell proliferation. Given the dependency of UPS models on glutamine, the therapeutic effects of telaglenast (CB-839), a small molecule GLS inhibitor, were evaluated in vitro and in vivo. Histological analysis of established murine tumours showed that the drug increased cell cycle arrest, decreased cell proliferation and increased cell death which was accompanied by a reduction in tumour size when compared to controls.

In another study, Lemberg et al. showed that malignant peripheral nerve sheath tumours (MPNST) were highly dependent on glutamine as MPNST cells required higher glutamine concentrations post-glutamine starvation to rescue their growth compared to healthy peripheral cells and immortalized Schwann cells [45]. They found that a glutamine antagonist JHU395 significantly inhibited tumour growth compared to vehicle treatment in vivo. To evaluate the effects of JHU395 on glutamine-dependent biosynthesis, tumour metabolomics was carried out by mass spectrometry. Out of 400 metabolites analysed, 18 had a statistically significant fold change between vehicle and JHU395-treated tumours. Multiple pathways involved in amino acid metabolism, nucleotide (purine and pyrimidine) synthesis and hexosamine synthesis were dysregulated. The most significantly altered metabolite was formylglycinamide ribonucleotide (FGAR), a precursor in de novo purine biosynthesis. Collectively, these two studies indicate that exploiting glutamine metabolism either through targeting of enzymes such as GLS or by using glutamine antagonists may have utility in the treatment of selected STS subtypes.

Arginine is a semi-essential amino acid, obtained from dietary sources and synthesized via the urea cycle in the kidneys [46]. One of the predominant metabolic changes reported in various cancers is the lack of expression of arginine succinate synthetase 1(ASS1). ASS1 is a rate-limiting enzyme required to convert citrulline to arginine, and its deficiency results in arginine auxotrophic cells, i.e. dependent on extracellular arginine for survival. In sarcomas, Bean et al. showed that IHC analysis of 701 tumours across 45 histological subtypes identified the absence of ASS1 in 90% of the samples [47]. Arginine deprivation in ASS-deficient cells led to starvation and reduction in cell survival. ADI-PEG20 (pegylated arginine deiminase) is able to reduce extracellular arginine resulting in prolonged arginine starvation and metabolic stress. The authors further showed that a combination of ADI-PEG20 with chloroquine enhanced metabolic stress resulting in cellular apoptosis and necroptosis. In a follow-on study by the same group, Kremer et al. undertook metabolic profiling to determine the global effects of acute and chronic arginine depravation in ASS1-deficient cell lines [48]. By utilizing LMS cell lines that have undergone short- or long-term treatment with ADI-PEG20 and capillary electrophoresis mass spectrometry (CE-MS), they identified dramatic metabolic remodelling upon acute and chronic arginine starvation which includes alterations in key intermediates of the TCA cycle (citrate, succinate, malate, α-ketoglutarate), amino acid metabolism (glutamine, serine, glycine) and glycolysis. Using a multi-pronged approach including metabolic tracing experiments, the authors uncovered a range of different vulnerabilities that could be exploited in combination with ADI-PEG20 to enhance tumour cell death. This included targeting upregulated glutamine metabolism through the use of GLS inhibitors and the utilization of anti-folate metabolites such as methotrexate as a means to exploit the diversion of glucose into serine biosynthesis and downstream single-carbon folate metabolism. A phase 2 trial (NCT03449901) evaluating ADI-PEG20 in combination with gemcitabine and docetaxel in STS is ongoing [49].

Tryptophan (Trp) is an essential amino acid, and a small fraction of its free levels in the body is utilized for protein synthesis and production of certain neurotransmitters and neuromodulators [50,51]. The remaining majority of Trp (over 95%) serves as a substrate for the kynurenine (Kyn) pathway, which is one of the major catabolic routes for tryptophan metabolism leading to the production of nicotinamide adenine dinucleotide (NAD) [52]. For this reaction to proceed, tryptophan undergoes oxidation to form kynurenine by the enzymatic activity of either indoleamine 2, 3- dioxygenase1, (IDO1), IDO2 or tryptophan-2, 3-dioxygenase (TDO2) [53]. This is an important rate-limiting step for extracellular Trp depletion with multiple key organs such as the intestine, brain, liver and immune cells serving as sites for Trp metabolism. Imbalances in the levels of Trp and its metabolites have been implicated in numerous disorders such as autoimmune and neurodegenerative diseases as well as cancer. There is a strong link between IDO, cancer and immune cell regulation, with small molecule inhibitors of this enzyme alone or in combination with immune checkpoint inhibitors under consideration for cancer therapy [54]. Preclinical data have shown that IDO1-deficient mice are more sensitive to immune checkpoint inhibitors such as anti-CTLA4, anti-PD1 and anti-PDL1 [55].There is also evidence that Trp depletion leads to induction of T regulatory cells, downregulation of T cell receptor chain expression in CD8+ cells and induction of inhibitory receptors in dendritic cells, all facilitating tumour immune escape [56].

In a single-arm, phase 2 multicentre study with 57 advanced STS patients (NCT02406781) comprising mostly LMS and UPS subtypes (54% of the cohort) evaluating the safety and efficacy of the anti-PD-1 antibody pembrolizumab in combination with metronomic cyclophosphamide [57], it was shown that a significantly higher Kyn to Trp plasma ratio was seen in patients during treatment. In a follow-up preclinical study, the same group evaluated the effects of Kyn pathway enzymes upon PD-L1 inhibition in syngeneic models of sarcoma [55]. PD-L1 blockade resulted in an increase in enzymes involved in Kyn pathway including IDO1 and IDO2 alongside inflammatory cytokines. Next, the authors investigated the impact of an IDO inhibitor (GDC 0919) in preclinical sarcoma models either as a single agent or in combination with the anti-PD-L1 antibody. This resulted in a significant decrease in plasma Kyn to Trp ratio but unfortunately had no anti-tumour activity. More preclinical and translational research is therefore required to dissect the interaction between the Trp metabolism pathways with immune cell regulation in order to improve the activity of immune checkpoint inhibitors in STS.

## Conclusion

Taken together, these studies demonstrate the power of proteomics and metabolomics to generate new hypotheses for biological interrogation, novel therapeutic candidates for drug discovery, previously undescribed molecular subgroups within histological subtypes and predictive and prognostic biomarkers. In some cases, these findings have been translated into clinical trials or have offered insights into why some therapies have failed in the clinic. Coupled with the latest technological innovations in mass spectrometry and imaging approaches, including new methods for single-cell analysis, it is anticipated that further discoveries in STS will be forthcoming.
